# Evaluating mobile app performance through sentiment analysis with SimCLR and MobileBERT

**DOI:** 10.1371/journal.pone.0349555

**Published:** 2026-06-01

**Authors:** Ruping Zhang, Aman Ullah, Khair Ullah Khan, Muhammad Nawaz Khan, Rehan Tariq Chohan, Farhan Aadil

**Affiliations:** 1 Department of Mathematics and Information Engineering, Liaocheng University, Dongchang College, Liaocheng, Shandong, China; 2 Department of Higher Education, Archives and Libraries Khyber Pakhtunkhwa, Peshawar, Pakistan; 3 University of Science and Technology, Bannu, Khyber Pakhtunkhwa, Pakistan; 4 Department of Smart Security, Gachon University, Seongnam-si, Gyeonggi-do, Republic of Korea; 5 Faculty of Modern and Technological Sciences, The National University of Malaysia, Qatar Campus, Lusail, Qatar; 6 Computer Engineering Department, Sivas University of Science and Technology, Sivas, Turkey; Sri Venkateswara University College of Engineering, INDIA

## Abstract

Mobile apps have significantly enhanced user engagement and convenience, providing seamless access to services anytime, anywhere. This paper presents a sentiment analysis (SA) model comprising SimCLR (Simple Contrastive Learning of Representations) and MobileBERT to measure the perceived performance of the mobile apps. The reviews of mobile apps are manually labeled as High and Low based on performance parameters (insightfulness, transparency, pertinence, precision, consistency, elaboration, and practical usability insights). These labels are aligned with attitudinal components, i.e., appreciation and judgment from appraisal theory. Appreciation concerns insightfulness, transparency, and pertinence of mobile app features, while judgment pertains to precision, consistency, elaboration, and practical usability. By integrating appraisal theory into this proposed model, a more comprehensive insight into user sentiment is achieved as compared to traditional SA models, which are heavily dependent on a very limited set of sentiment categories, i.e., positive and negative. The accuracy of manual annotation is ensured by the SHAP (SHapley Additive exPlanations) method of Explainable AI (XAI). In this study, SimCLR, a self-supervised learning framework, is employed to improve feature extraction and enhance the model’s ability to generalize across different datasets, while MobileBERT, a lightweight transformer model for sentiment classification, ensures computational efficiency and high performance. The MobileBERT makes the proposed approach scalable, efficient, and highly suitable for real-time SA in resource-limited environments. Knowledge distillation is incorporated to teach a student model that is similar to the teacher model, increasing the robustness of the system. In this work, the Augmentation of data by replacing synonyms in the data using WordNet increases the quantity of the training data to make the model more robust to linguistic variations. The proposed model is optimized using the PCA (Principal Component Analysis) method for dimensionality reduction. The Optuna method is used for hyperparameter optimization, automating the search for the most effective configuration, including learning rate, batch sizes, etc. K-fold stratified cross-validation ensures model robustness. The results demonstrated that the integration of SimCLR, MobileBERT, distillation, data augmentation techniques, and fine-tuning using Optuna provides a highly efficient and accurate SA-based model, with a mean fold accuracy and ROC AUC of 88.63% and 90.91%, respectively. This approach offers a scalable solution for NLP (Natural Language Processing) tasks. The proposed approach will be highly beneficial not only for the developers, yielding a deeper and nuanced understanding of the user feedback to identify specific areas for improvement in app performance, but also to stakeholders like marketers, product managers, and user experience (UX) researchers.

## Introduction

There are millions of apps available in different categories such as finance, banking, games, social media, and healthcare, etc. The success of apps is affected by many factors; one crucial factor is online reviews. Consumers are often looking at customers’ feedback from app stores when deciding which apps to download [1]. After users download and install these apps, they are often asked to leave ratings and reviews based on their experience. These ratings and reviews are extremely important, as they help other users to decide whether to install the app or not. In their reviews, users give their views about things such as how easy the app is to use, how well it performs, its features, and any issues they encountered [2]. The opinion is much more detailed in its description of the user’s experience and gives context and specific feedback. While ratings do give a kind of snapshot of satisfaction levels and are fast, they don’t really give a whole lot of insight into how the app can be improved. Reading the opinions in the reviews allows for greater comprehension and better clarity of what users like and dislike. But there’s no consistent format on how users will write their text-based reviews, which makes it more challenging to analyze the data of reviews, as they are more disorganized, making it more difficult to interpret [3].

The Google app market collects opinions of users in the form of ratings and textual reviews based on their thoughts and satisfaction with an app. The feedback offered in these reviews helps other potential users decide on their choice to download or purchase an app. However, manually analyzing all the reviews is not practical because of the sheer number of reviews. However, instead, SA, which uses machine learning (ML) algorithms and NLP, is used to analyze these reviews on an automated basis [4]. The information in apps’ reviews assisted the developers in developing other apps to fulfill the requirements of users and also make their products successful [5]. The authors in [6] proposed an aspect-based SA (ABSA) model to extract the perceived quality of an app by applying the techniques of ML and deep learning (DL). They established that ML models, especially the LR using the BERT embeddings, were effective in the ABSA task in comparison to the DL models. In [7], the authors have suggested three prioritization approaches related to the problem of app review analysis, which also covered the gap of prioritizing issues for the evolution of apps. These approaches relied on four attributes, namely frequency, rating, negative emotions, and deontics, which help the developers to focus on the most critical improvements.

In [8], the authors explored the connection between the characteristics of the devices and applications with the quality of Android applications as rated by users. Their results indicate that the device attributes, such as CPU, are significant determinants of user ratings, as well as the app attributes, such as code size. Their study highlights that manufacturers and developers should pay attention to both the features of the device and the application in order to maximize the effectiveness of the app and its acceptability by the users. In [9], the authors conducted a review of related work on the solutions to help mine online opinions in app store reviews. They pointed out the increasing significance of user feedback to developers and business. B. Chembakottu [10] carried out a detailed survey regarding the perception of mobile apps quality among users driven by Android Sports applications. In his study, the author examined the implications of various dimensions in user reviews and rating, including the app categories and functionalities, and revealed some insights within sports apps.

In this study, we introduce an efficient SA-based framework that aims to handle these challenges. The framework is based on a SimCLR-based architecture using MobileBERT as a backbone, which is based on a self-supervised learning method with a lightweight transformer model for sentiment classification. The model improves the feature extraction of the model using SimCLR, and computational efficiency is ensured using MobileBERT. Knowledge distillation is included to train a strong student model to mimic the teacher model. To make the proposed model more robust against the variation in the language, the data augmentation method with the help of WordNet is used that augment the apps’ reviews for the model’s training. This approach is optimized using the PCA method for dimensionality reduction and Optuna to optimize hyperparameters, which finds the most effective configuration automatically. K-fold stratified cross-validation is used to validate the robustness of the model. All of the above components are integrated into a powerful and effective sentiment analysis framework, providing a unique solution to the problem of sentiment classification in mobile application reviews. This architecture is a novel combination of techniques and is scientifically underpinned to provide improvements in sentiment analysis models, not only in accuracy but also running capability.

### Contributions

The key contributions of this work are as follows.

- To include manually annotating the reviews based on the appreciation and judgment of components of the appraisal theory and making a categorization of it into “High” or “Low” according to predefined sentiment criteria.
- To ensure the accuracy of the manual annotations, the SHAP is used, giving transparency to make efficient decisions about the proposed model and validating the manual annotations by assigning values to the importance of the features to ensure alignment with human-understandable reasoning.
- To improve feature extraction further, the paper also incorporates SimCLR, a self-supervised learning method better able for generalization across different datasets so that the model improves its ability to adapt to a variety of mobile app reviews.
- In order to do good sentiment classification, MobileBERT is where computational efficiency is the focus, which is especially the case in scenarios with limited computing resources, such as mobile devices.
- To further improve generalization, the approach of knowledge distillation takes place, where a smaller model called a student learns to mimic the predictions of a larger model called a teacher, to improve the overall performance of the model.
- To make the model robust to the linguistic differences, data augmentation by the form of replacing the words with their synonyms using WordNet is injected, so that the model is robust enough to capture diverse expressions of sentiment from user-generated content.
- To optimize the proposed framework, Optuna is used for hyperparameter tuning, which helps in automatically estimating the best configurations for the network, such as rate of learning, batch size, weight decay, size of epochs, etc., in order to ensure the best model performances.
- To process the reviews, the ReviewDataset class is used, in which the reviews are tokenized using MobileBertTokenizer, and augmentation of reviews are prepared using synonyms, and for handling imbalanced dataset, weighted sampling using DataLoader is utilized.
- To validate the robustness of the model, K-fold stratified cross-validation is used, which ensures each fold has the same representation of high as positive and low as negative reviews.

## Related work

Mobile app reviews help users to share their feedback and improve the quality of the apps, and developers use mobile app reviews to enhance their apps using real-world experiences [11]. Researchers know how critical user eXperience (UX) is in mobile app development and getting past usability to provide a better user experience [12]. The authors in [13] created a new framework with ML models such as AdaBoost, XGBoost, and ANNs to assess app rating sentiments available in the Google Play Store. By using a large database from GitHub, they were able to optimize their approach, resulting in 98% precision and 85% recall, surpassing the results from previous studies. The authors in [14] discussed user perceptions, app reputation, and price-level issues which were overlooked in traditional Apps. In [15], the authors introduce UX-MAPPER, a software to identify factors that affect User eXperience (UX). The tool was made with the design science research method, and testing was carried out with exploratory, systematic mapping and empirical studies. Participants saw the relevance of UX-MAPPER in raising the quality of apps and gaining an understanding of user preferences, although feedback indicated that further refinement is required to improve the quality of the output produced for experienced practitioners. The authors in [16] evaluated support vector machine (SVM) and naive bayes (NB) to conduct SA of TikTok app reviews. Their findings indicate that the performance of SVM was better than that of NB.

In [17], the author identified the users’ sentiments of the TJ: Transjakarta application reviews. Sentiment labels were determined by a dictionary-based approach. The accuracy result of the IndoBERT-base-p1 model was 87.76%. H. P. Wijaya et al. [18] pointed out the development of digital technology, which resulted in more usage of the JMO application that has gained many reviews in the Google Play Store. They used the BERT model for SA on these reviews and classified the reviews into positive, negative, and neutral ones. The results demonstrated that the accuracy increased from 0.60 to 0.65 when the BERT model was fine-tuned and achieved the highest F1-score of 0.80 for positive sentiment. In [19], the authors analyzed feedback on the Wargaku Surabaya application, to categorize emotions into different After comparison of NB and SVM models, it was found that SVM’s performance was higher than NB. In [20], the authors suggested Wiscom, a tool that helps to analyze millions of user evaluations on mobile application marketplaces like Google Play Store. Wiscom provides insights at three levels: (a) discovering inconsistencies in reviews (b) discovering the reasons why users like things and (c) providing information on the market at large about what users worry about and which apps they most prefer. The tool has been evaluated using in a dataset consisting of more than 13 million reviews covering 171,493 of Android apps. By providing outside assistance to the app market perators, developers and users using it helps them find more valuable sources of information.

In [21], the authors examined more than one million reviews from 800 Android apps to provide a measure of user satisfaction with Augmented Reality (AR) and Virtual Reality (VR) in education. Using BERT to work on SA they get positive sentiments of 54.6% for AR, 49.6% for AR+Educational, 47.7% for VR, 71.4% for VR+Educational and 75.2% for Educational apps. The study suggested that educational apps without AR or VR are given higher user satisfaction indicating some key elements for improvement when using AR/VR based apps. In [22], the authors explored feature selection methods to use the combination of Information Gain, GINI Index and Correlation Matrix to enhance the ML models for app user reviews. They obtained an F1-Score of 80.81% with the SVM Model that highlights the importance of quality features for multi-label classification tasks. The authors in [23] set forward an XAI approach utilizing BiLSTM-based model and attention mechanism to help mobile app developers prioritize feature requests from user reviews. Susanto et al. [24] focused on SA of reviews for the XYZe-wallet application which has a low rating of 2.7. Using Word2Vec and LSTM model, they were able to achieved high accuracy and visualize the learning using BERTopic and BART to solve the balance top-up problem in product improvements. The authors in [25] proposed a method of prioritizing the user reviews of Google Play, which addresses the issue of responding efficiently to reviews. By extracting textual and semantic features and training 4 ML models, they found that the XGBoost ML model was the best to identify which reviews needed responses.

In [26], the authors discussed the concept of mining Non-Functional Requirements (NFRs) from user reviews of mobile app stores and how this is important in achieving user satisfaction. In the first phase, a qualitative analysis of 6000 reviews revealed that the NFRs showed by 40%, with different categories of apps raising different types of NFRs. In the second phase, an optimized dictionary-based multi-label classification approach was proposed with an average precision of 70% and recall of 86% on 1,100 reviews from iOS and Android apps. The authors in [27] evaluated user perceptions about the quality of mobile application services using Google Play reviews in OTA applications such as Traveloka and Tiket.com. By using SA and topic modeling, they demonstrated positive and negative sentiments and key topics concerning the mobile application service quality dimensions. In [28], the authors created a quality assurance process for mobile apps. light-weight analyses using user feedback to support the decision of the product managers. Their approach involves the inclusion of emojis to extract emotions, detecting trends and generating ideas to improve them with examples from popular apps. In [2], the authors used ML models to apply SA of user reviews of mobile applications from the Google Play Store. Using random forest (RF), logistic regression (LR) and K Nearest Neighbours (KNNs), they concluded that LR model performed better, providing 95.15% accuracy compared to the other models, which represents the importance of model selection and feature engineering in SA for app developers and users.

The authors in [29] emphasized the increasing attention of mobile app analytical firms on the development of data analytics to undertake competitive analysis and market research. Their study by tracking user interactions and analyzing app-centric metrics predicts user ratings with DL models and ML models such as Naive Bayes, XGBoost, and MLP. Mahmood, Toqeer, et al. [30] discussed the use of smart devices and the Covid-19 pandemic that has impacted the widespread use of the mobile banking application, which have improved the efficiency of banking and living standards. Using ML classifiers and Top2Vec model, they performed SA analysis of 10 Pakistani mbanking apps and identify positive themes such as ease of use and negative themes such as performance. The ensemble model has proved to be the better model with an f1-score of 90%. In [31], the authors reviewed 104 mental health apps using user reviews (88,125) with SA and ML models, when looking for positive or negative themes. In [32] the authors discussed the utilization of ABSA to automate the process of analyzing user reviews of mobile applications. By fine-tuning a BERT-based ABSA model, they extracted reviews with sentiment triplets (aspect, opinion, polarity) and showed the effectiveness of ABSA in obtaining feedback regarding app features. The proposed framework when combined with Topic Modeling, gives developers actionable insights to better the user experience. Using NB algorithm for sentiment classification, the reviews of Coursera application were translated to the Indonesian language by the authors in [33] under the Knowledge Discovery in Databases (KDD) approach, they found that translation did not have significant effect on the main sentiments: positive ones were concerned with how good the quality of courses was, and technical problems pushed negative feedback.

The authors in [34] examined user sentiment about the “Access by KAI” application, as they were interested in aspects such as speed, payment process, and UI/UX. User reviews were processed using SVM, Decision Tree and LR, whereby SVM model got the highest accuracy of 89.33%. In [35], the authors suggested a new pattern in predicting outliers between numeric ratings and text reviews on the Google Play Store, which solved the problem of fake rating. The framework was divided into two stages – polarity of review was predicted by SA, and then the star ratings were predicted by reviews using the DL models. The authors in [36] used text mining methods such as LDA Topic Modeling and SA to analyze 230,940 reviews of nine mobile payment apps. They identified four key factors – ease of payment, security, and customer service, and app design – underlining the huge influence of good and bad ratings on app performance. In their study, the authors also discussed the sentiment analysis (SA) of user reviews to extract requirements for enhancing mobile applications. They highlighted the importance of SA in interpreting user feedback and using automatic approaches to derive features and sentiments. In [37], the authors proposed a framework for performing a SA of tweets at both the message and term level using supervised statistical classification and tweet-specific sentiment lexicons. The system was ranked 1st in the SemEval-2013 shared task with an F-score of 69.02 on the message level and 88.93 on the term level task.

In [38], the authors suggested a model based on DistilBERT in order to predict the quality of mobile apps by classifying user reviews based on the high or low quality, with include linguistic indicators such as informativeness, clarity and usability feedback. It involves bringing together the main elements of the appraisal theory, such as affect, appreciation, and judgment to achieve more insights into the evaluation provided by users. An explainable AI (XAI) solution (SHAP) is applied to guarantee the interpretability of a model, which validates whether the model is focused in areas with core quality measures, such as usability and stability. In [39], the authors examined the sentiment and topics of review content from 25 digital detox apps in the Google Play Store by applying the SA and LDA topic modeling methods. Positive sentiments, particularly ‘trust’ and ‘anticipation’ were frequent and regression analysis showed that sentiment scores were correlated with app ratings. This research is of great help to the developers to improve the digital detox apps by a new approach that uses text mining, SA, and topic modeling. The authors in [22] used feature selection techniques such as Information Gain, GINI Index, and Correlation Matrix to enhance the ML models to analyze app user reviews. The authors in [40] examined consumer reviews of m-banking apps from five banks in Canada based on their data using SA and topic modeling techniques to determine user preferences and areas for improvement. LSTM provided 82% accuracy for iOS reviews while NB has provided 77% accuracy for Google Play. Positive reviews commented on usability while negative reviews highlighted login problems and glitches. The SA of the reviews of the Spotify app was presented via NLP, using transformer-based models such as BERT, DistilBERT, RoBERTa, and XLM-RoBERTa by the authors in [41]. DistilBERT had the highest accuracy score (71.68%) and XLM-RoBERTa the best F1 Score (69.24%).

In [42], the authors examined the user sentiment towards mobile banking applications in Indonesia using the RF, KNN, NB, and SVM algorithms and the effectiveness for each of these algorithms. Reviews from 4000 samples was taken from Google Play Store was preprocessed by stopword removal and tokenization using the Bag of Words method. RF was the best predictor with accuracy of 0.58 and 0.74 for BCA and Brimo assessment, respectively, whereas the accuracy of NB and SVM was better than the other algorithms for BNI and Beyond by BSI applications, respectively. In [43], the authors utilized the SVM algorithm for analyzing the user’s sentiment towards the X app on Google Play Store by processing 5,000 reviews by cleaning, tokenization and sentence reconstruction. Sentiments were divided into neutral, negative, and positive categories SVM had shown 86.3% accuracy. The neutral class performed the best in terms of recall with a value of 0.96, the negative class in terms of precision with a value of 0.95, and the positive class in terms of F1-score with a value of 0.78 because of imbalanced data. The authors in [44] implemented a pre-trained BERT model for performing a refined SA on Play Store app reviews, solving the limitations with tokenization, fine-tuning, and hyper-parameter optimization. The model achieved 84.3% accuracy and an F1-score of 0.84, which outperformed traditional ML models. Similarly, the authors also created a user review SA model for a Play Store app like GoPay by comparing text representations from TF-IDF and BERT algorithms with RF and LR algorithms. The combination of BERT and LR managed to score the best with an F1 Score of 0.86, which shows the superiority of BERT in understanding the semantic context.

After thoroughly studying the existing work, it reveals that the existing studies mainly emphasized the conventional ML algorithms or naive transformer models, which do not provide nuanced semantic insight and shallow feature learning. Also, explainability and real-time computational efficiency in resource-constrained environments are not given much attention. Mobile app sentiment analysis incorporating the theory of appraisals, contrastive learning, and knowledge distillation has not been explored thoroughly. Moreover, the majority of the models do not integrate state-of-the-art methods, such as data augmentation, dimensionality reduction, and hyperparameter optimization, in mobile apps’ SA. This raises a deficiency of a more powerful and semantically extended SA model, which can provide a deeper insight and enhanced performance. Comparative analysis of related work is shown in Table 1.

**Table 1:** Comparative analysis of related work.

Reference	Model/Technique	Key Features	Limitations
[13] M. Q. Khan et al., 2024	AdaBoost, XGBoost, ANNs	98% precision and 85% recall for sentiment analysis	Lacks advanced feature extraction and deep semantic understanding
[14] A. Al-Subaihin et al., 2015	App Store Mining	Focuses on app store data analysis	No sentiment analysis
[15] W. T. Nakamura et al., 2025	UX-MAPPER Tool	Automates UX review analysis	No sentiment analysis capability
[16] J. O. Leandro et al., 2025	SVM vs. Naïve Bayes	SVM outperforms Naïve Bayes	Limited semantic understanding
[17] N. A. Putri et al., 2026	IndoBERT	87.76% accuracy (dictionary-based)	Limited semantic depth
[18] H. P. Wijaya et al., 2026	BERT	Up to 80% F1-score	Not efficient for real-time
[19] L. N. Fadilah et al., 2026	SVM vs. Naïve Bayes	Improved classification with SVM	Limited semantic capability
[20] B. Fu et al., 2013	Wiscom Tool	Extracts user review insights	No sentiment analysis
[21] A. S. Mondal et al., 2024	BERT (AR/VR)	Sentiment analysis for AR/VR apps	Limited semantic depth
[22] A. Salleh et al., 2025	SVM + Feature Selection	Improves efficiency via feature selection	No advanced feature extraction
[23] I. Gambo et al., 2024	XAI + BiLSTM	Explainable prioritization of feedback	Weak sentiment capability
[24] Susanto et al., 2025	Word2Vec + LSTM	High accuracy with embeddings	Limited flexibility
[25] M. Jafari et al., 2025	XGBoost	Identifies critical reviews	No sentiment analysis
[26] N. Jha et al., 2019	NFR Mining	Extracts non-functional requirements	Dictionary-based limitation
[27] R. A. Masrury et al., 2019	OTA Sentiment Analysis	Evaluates service quality	No transformer models
[29] S. Venkatakrishnan et al., 2020	ML/DL Analytics	Analyzes app metrics	Limited semantic depth
[30] T. Mahmood et al., 2023	ML + Top2Vec	Sentiment analysis for banking apps	Not optimized for semantics
[31] O. Oyebode et al., 2020	ML Models	Mental health review analysis	Lacks deep semantics
[32] V. Ballas et al., 2024	ABSA with BERT	Aspect-based sentiment analysis	Limited semantic depth
[33] M. Rizqi et al., 2025	Naïve Bayes	Sentiment classification	Limited flexibility
[34] P. T. Prasetyaningrum, 2025	SVM	Sentiment classification	No deep semantic modeling
[42] S. Rahayu et al., 2025	RF, SVM, KNN	Traditional ML approaches	Lacks deep semantic analysis

### Problem statement

Existing studies have not employed the components of appraisal theory in the manual annotation process which are essential for the proper analysis of the nuanced component of sentiment in user reviews. Traditional forms of SA typically relied on polarity, i.e., positive and negative only, or on some form of simple keyword-based methods. Moreover, in many models there are no guarantees for accuracy in annotations or transparency in the process by which the model arrives at a decision. To compute the performance of mobile apps, there is a shortage of an efficient and computationally inexpensive hybrid DL-based framework. This study addresses these gaps by considering the elements of appraisal theory for manual annotation, and validates the annotations using SHAP (SHapley Additive exPlanations) from XAI for accuracy and interpretability. The framework also utilizes a combination of SimCLR, MobileBERT, knowledge distillation and data augmentation to improve the model’s performance, robustness and scalability and offer a comprehensive solution for SA of mobile apps reviews.

## Methodology

This section comprises of a description on the dataset, an annotation example on using Appraisal Theory, data preprocessing steps, the SHAP method, a detailed explanation of the proposed model and its corresponding pseudocode.

### Dataset

The proposed model in this paper is evaluated with 19,278 reviews [45] of 7 well-known mobile applications as shown in Table 2. The reviews were fetched by using the Google Play Reviews API and processed using Jupyter Notebook.

**Table 2:** Description of dataset.

App Name	Number of Reviews
Daraz Online Shopping	907
ESPNcricinfo	4796
MX Player	993
Opera Mini: Fast Web Browser	3194
Skype for Business for Android	5284
Snapchat	3228
VLC for Android	876
**Total**	**19278**

### Data annotation

In this research work, the mobile app reviews are annotated by a group of trained researchers who have knowledge of sentiment analysis. In order to make the annotations reliable and consistent, we carried out an inter-rater agreement check. In this study, the Cohen Kappa rule is employed to measure the consistency and accuracy among annotators’ work. This process guaranteed reliability and consistency of the annotations, which gave a good ground on which to train ML models. With this approach, we ensured a high level of reliability of the annotation, which is also essential to the validity and efficiency of the sentiment analysis model.

### Examples of data annotation

Table 3 comprises of mobile app reviews and its annotations using two major aspects of appraisal theory – appreciation and judgment. Appreciation is about the good qualities of the app, as in its functionality, aesthetics or reliability while judgment is about app’s general performance and the user’s experience, which may include some areas where they can be improved. The sentiment column classifies each review to either high or low depending on the sentiment expressed in the feedback. These reviews are from popular mobile apps which includes -espn, snapchat, skype, opera mini, mx player, vlc mediaplayer, daraz online shopping etc. These reviews are meant to illustrate the range of user perceptions regarding different features in the apps.

**Table 3:** Data annotation examples.

App Name	Review	Appreciation/Judgment	Sentiment
ESPNcricinfo	The app provides accurate sports updates and live scores.	Appreciation (functional effectiveness)	High/Positive
Snapchat	Love the filters and fun interactions, but it crashes often while sending snaps.	Appreciation (aesthetic value)	Low/Negative
Skype for Business for Android	Video quality is great, but connection drops frequently during calls.	Judgment (performance evaluation)	Low/Negative
Opera Mini Fast Web Browser	Provides fast browsing and efficient performance.	Appreciation (functional effectiveness)	High/Positive
MX Player	Sometimes playback lags on certain videos.	Judgment (quality of user experience)	Low/Negative
VLC for Android	Reliable media player that supports multiple formats efficiently.	Appreciation (reliability)	High/Positive
Daraz Online Shopping	Fast delivery and wide product range, but customer support needs improvement.	Judgment (customer service evaluation)	High/Positive

### Data preprocessing

In the proposed model, we performed a number of data preprocessing steps for enhancing the quality of app reviews and for preparing the data for ML analysis. First, we would have performed tokenization to segment each review sentence into smaller tokens, with some delimiters including semicolons, commas, and periods. Next, we changed the text to lower case, so that it is uniform and there are no discrepancies of case sensitivity. We then removed the punctuation, as punctuation doesn’t have significant semantic value, and will add noise into the data. Following this, we removed stop words such as “the,” “is” and “and” that are not meaningful for any SA, so that we can concentrate on more meaningful words. For example, a review like “The app is very slow and crashes often” was tokenized as [“app,” “slow,” “crashes,” “often”] with the help of stop words removed to emphasize what made the app being reviewed so great.

### Detailed description of proposed model

The proposed approach is developed in two separate phases, which mainly focus on different components. In the first phase, manual annotation in respect to the perceived performance of apps’ reviews is performed. The predefined performance indicators are based on keywords and phrases that are commonly associated with positive or negative sentiments in user reviews.

Once the reviews are manually annotated, the annotations are validated using SHAP from Explainable AI (XAI), which provides an interpretability layer on top of the sentiment model’s predictions. SHAP assigns feature importance values to the words and phrases present in the reviews to provide transparency, which assists the proposed model in making efficient decisions. This validation through SHAP ensures the accuracy of the manually labeled data. The SHAP values also give insight into the influence of the different features (words or phrases) as to what can influence whether a review is considered “High” or “Low”. In the second phase, we introduce advanced NLP techniques in order to improve the model performance and generalization. The model architecture uses SimCLR to extract important features from the data. SimCLR helps the model generalize better across different distributions of mobile app reviews so that they become more robust to different styles of reviews and content. MobileBERT, a lightweight version of the BERT model, is used to optimize the fine-tuning of a classification head in order to accomplish SA while maintaining a high computational efficiency, especially in mobile environments with limited resources.

In this study, the teacher model is a pre-trained SimCLR MobileBERT model, a powerful feature extractor of textual data specifically designed to deal with the complexities of sentiments of reviewing an app. This model is not a fine-tuned model but is applied to produce soft target probabilities used to train the student model. The student model is a fine-tuned sentiment mobileBERT model, highly tailored to predict sentiment labels (positive or negative) of reviews of mobile apps. The student model is trained with knowledge distillation, where the DistillationLoss of KL divergence is between the soft probabilistic predictions of the teacher model and the hard prediction of the student model as well as the standard cross-entropy loss on the true sentiment labels. The method enables the student model to learn effectively with the labeled data (i.e., app reviews with sentiment labels), as well as with the knowledge coded in the teacher model and thereby improves its accuracy in the sentiment analysis tasks without generalizing to other app reviews.

**Fig 1:**
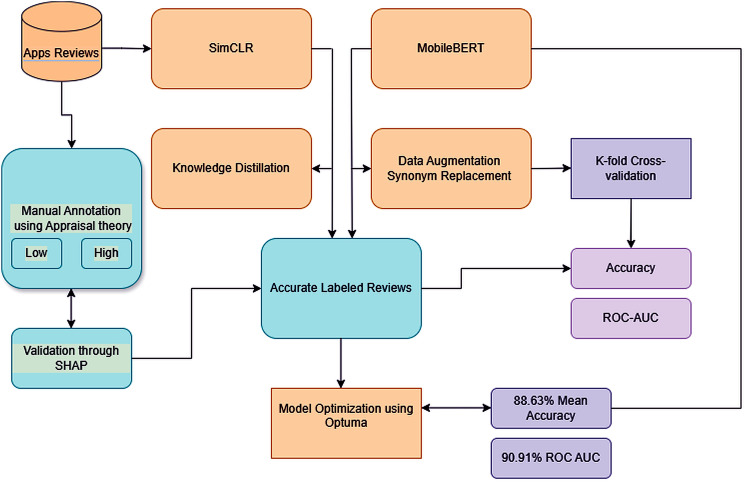
Proposed model to evaluate the performance of mobile apps.

Data augmentation is also implemented in the form of synonym replacement using WordNet, which boosts the diversity of data used for training and ensures that the model is robust and does not get confused with any linguistic variations in the user-generated content. Optuna method is employed for hyperparameter tuning of parameters like learning rate, etc. Hyperparameters are sampled, like the learning rates from 1e-5 to 1e-4, batch sizes from 16, 32, 64, weight decay from 0.01 to 0.1, and epochs from 3 to 5, which maximize the ROC AUC score of the model. The dataset is processed using the Review Dataset class, which includes tokenization of the reviews using MobileBertTokenizer and augmentation with the help of a synonym. The performance of the model is tested by K-fold stratified cross-validation, and an equivalent amount of positive and negative reviews will be used in each fold. After the model is trained and tested across 5-fold cross-validation validation, the results in the form of mean fold accuracy of 88.63% and the ROC AUC of 90.91% are achieved, which shows the effectiveness of the hybrid model (SimCLR, MobileBert, knowledge distillation, data augmentation, and Optuna for hyperparameter optimization). This approach offers a scalable, efficient idea for SA that can deal with the linguistic diversity of user reviews, with high accuracy. The proposed model, as shown in Fig 1 and its pseudocode, is represented as Algorithm 3.

**Algorithm 1. Performance evaluation framework for mobile application reviews.**

**Input:** Mobile application reviews dataset ℛ

**Output:** Mean classification accuracy and average ROC–AUC

**Phase I: Manual Annotation and Explainability Validation**

1. For each review r∈ℛ: a. If *r* satisfies predefined performance indicators, assign sentiment label = High. b. Otherwise, assign sentiment label = Low. c. Store the manually annotated review.

2. Apply SHAP to compute feature importance scores.

3. For each review r∈ℛ: a. Identify dominant features influencing the sentiment prediction. b. If SHAP-based validation confirms annotation consistency, approve the annotation.

**Phase II: Representation Learning and Model Optimization**

4. Extract semantic representations using SimCLR.

5. Initialize MobileBERT and perform task-specific fine-tuning.

6. Initialize teacher model (BERT-Large).

7. Train lightweight student model via knowledge distillation.

8. Enhance training data using synonym-based augmentation (WordNet).

9. Conduct hyperparameter optimization using Optuna.

10. Tokenize reviews and construct the training dataset.

**Phase III: Model Validation and Performance Assessment**

11. Perform 5-fold cross-validation: a. Partition the dataset into training and validation subsets. b. Train the optimized model. c. Evaluate performance using Accuracy and ROC–AUC. d. Record fold-wise results.

12. Compute overall mean Accuracy and average ROC–AUC.

13. Return final performance metrics.

### SHAP (SHapley Additive exPlanations) for explainability

SHAP is an explainable AI (XAI) methodology with its roots in the cooperative game theory. It is useful to explain the predictions of a model by assigning the contribution of each feature to the final prediction. In particular, SHAP estimates the role of each word (or feature) in the review in predicting sentiment. The Shapley value for feature iii is calculated using the formula shown as Equation 1.


$$\begin{array}{c} \phi_{i}(v)=\sum_{S\subseteq N⧵\{i\}}\frac{|S|!\;(|N|-|S|-1)!}{|N|!}\left[v(S\cup \{i\})-v(S)\right] \end{array}$$ (1)

i) $\phi_{i}(v)$: This is the Shapley value for feature i.
ii) $\sum_{S\subseteq N⧵\{i\}}$: This represents the summation over all subsets S of the feature set N that exclude feature i.
iii) $\frac{|S|!\;(|N|-|S|-1)!}{|N|!}$: This is the weight applied to each subset, ensuring fair distribution of contributions.
iv) v(S∪{i})−v(S): This is the contribution of feature i, calculated by the difference in model predictions when feature i is included and excluded from the subset S.

### SimCLR (Self-Supervised Contrastive Learning)

SimCLR is a self-supervised learning model that is used to enhance the extraction of features by training a model to differentiate between similar and dissimilar data points. With respect to our case, the framework assists the model to learn powerful representations of the review text, which increases its capacity to be general to various datasets. The mathematical equation of the contrastive loss function used in SimCLR is shown as Equation 2.


$$\begin{array}{c} L_{\text{contrastive}}=-\log\left(\frac{\mathrm{sim}(z_{i},z_{j})}{T}\right) \end{array}$$ (2)

*L*_*contrastive*_: This represents the contrastive loss. It is a metric of the ability of the model to learn to differentiate between similar and dissimilar pairs. The smaller the contrastive loss, the better the performance, as the model is successfully finding or capturing similar entities that are near one another and dissimilar entities that are distant from each other.

$\log\left(\frac{\mathrm{sim}(z_{i},z_{j})}{T}\right)$: This is a similarity operation between two feature vectors, *z*(*i*,), and *z*(*j*). The value of similarity is logarithmicized, presumably due to the desirability of penalizing very low similarities more than high similarities. T is a temperature parameter, which is a scaling factor. It is used to regulate the similarity. Increased temperature results in a more uniform distribution of similarities, whereas decreased temperature results in the model paying more attention to very similar or very different pairs. This assists in optimization of the contrastive loss function.

### MobileBERT

MobileBERT is a thin and efficient transformer model. It employs a self-attention mechanism, which enables the model to attend to words in the review text that are important for prediction, irrespective of their position in the input data, thereby capturing contextual relationships between words. This is especially important for sentiment classification tasks, since words can often have different meanings based on their context. Self Attention Mechanism allows MobileBERT to capture contextual relationships between words in a sentence, regardless of their position. The self-attention mechanism used in transformers is mathematically described as Equation 3.


$$\begin{array}{c} A=\frac{QK^{T}}{\sqrt{d_{k}}}, \end{array}$$ (3)

Attention(Q, K, V) = Softmax(A) V,

where: i) Q, K, and V are the query, key, and value matrices, respectively.

ii) dk is the dimension of the key vectors.

### Distillation loss function

Knowledge distillation is harnessed in this work to provide a higher efficiency for the student model. Knowledge distillation transfers this “soft knowledge” from a larger teacher model to a smaller student model. Specifically, the distillation loss is a linear combination of two parts: 1) the Kullback-Leibler (KL) divergence between the outputs from teacher and student respectively and 2) the cross-entropy loss between predicted labels by student and true labels. The formula for distillation loss is shown as Equation 4.


$$\begin{array}{c} L_{\text{distill}}=\alpha \cdot \mathrm{KL}(P_{\text{teacher}}\;\|\;P_{\text{student}})+(1-\alpha )\cdot L_{\text{CE}}(P_{\text{student}},P_{\text{true}}), \end{array}$$ (4)

where: $P_{\text{teacher}}\;\|\;P_{\text{student}}$ are the softmax outputs in respect to the teacher and student models, respectively.

*L*(*CE*) is the cross-entropy loss and α is a hyperparameter that controls the balance between the KL divergence and cross-entropy loss.

In this way, the student model is forced not only to minimize classification error with respect to current labels but also to learn from more confident predictions made by teacher model, resulting in better generalization.

### PCA (Principal Component Analysis) for dimensionality reduction

Analogous, PCA is used to reduce the dimensionality of feature space by projecting the data onto the most critical principal components that account for maximum variance in the data. This approach reduces model complexity while maintaining critical information. The representation of the most significant features in a clear and understandable way is the benefit of PCA, which is in line with the purpose of enhancing the efficiency of the model in mobile environments when the resources are limited. PCA is more applicable to generalization in relation to a wide variety of data, including reviews of multiple mobile apps across platforms.

PCA is mathematically expressed as Equation 5


$$\begin{array}{c} X^{\prime }=XW \end{array}$$ (5)

Here X represents the original data matrix, and W is the concatenation of eigen vectors associated with components. PCA can be useful for visualizing high dimensional data in a lower dimension space, and is important for analyzing the model behavior.

### Hyperparameter optimization with optuna

By using Bayesian optimization, Optuna can efficiently and automatically search over the hyperparameter space (also known as hyperparameter tuning), such as learning rate and batch size. This guarantees that the model is trained with the best set of hyperparameters.

The loss valued minimized during optimiztion, is the objective function for Optuna and is represented as Equation 6.


$$\begin{array}{c} L_{\text{optuna}}(\theta )=L_{\text{val}}(\theta ) \end{array}$$ (6)

Where: where θ represents the hyperparameters and *L*_*val*_ is the validation loss. This optimization technique improves the model’s performance by fine-tuning the hyperparameters.

### Evaluation metrics

To evaluate the model’s performance, several metrics are calculated, including accuracy, F1-score, precision, recall, and a confusion matrix. These metrics provide a detailed understanding of the model’s classification performance. The entire process is implemented using PyTorch and PyTorch Geometric, with visualization of the results carried out using matplotlib and seaborn. Accuracy: Accuracy measures the percentage of correctly classified samples out of all samples in the dataset. The mathematical formula to calculate accuracy is shown as Equation 7.


$$\begin{array}{c} \text{Accuracy}=\frac{TP+TN}{TP+TN+FP+FN} \end{array}$$ (7)

**Precision:** Precision evaluates the model’s ability to avoid false positives (FPs). The formula for Precision is represented as Equation 8.


$$\begin{array}{c} \text{Precision}=\frac{TP}{TP+FP} \end{array}$$ (8)

**Recall:** Recall measures the model’s ability to capture all the true positives in a class. The mathematical equation to calculate Recall is represented as Equation 9.


$$\begin{array}{c} \text{Recall}=\frac{TP}{TP+FN} \end{array}$$ (9)

**F1-Score:** F1-score as shown in Equation 10, is the harmonic mean of precision and recall. It balances the trade-off between the two:


$$\begin{array}{c} \text{F1-Score}=2X\frac{\text{Precision}\cdot \text{Recall}}{\text{Precision}+\text{Recall}} \end{array}$$ (10)

**ROC-AUC:** ROC-AUC is just the ability of your model to differentiate between classes. TPR represents the rate of actual positives correctly predicted by the ML model, and FPR shows the rate of actual negatives that are falsely predicted by ML model as positive. ROC-AUC is the bottom section of the ROC curve that is used to measure the capacity of your model being able to differentiate between the classes. The mathematical equation to calculate ROC curves is shown as Equation 11.

**Let** *A* = TPR, *B* = FPR


$$\begin{array}{c} \int_{0}^{1}A\cdot B\;dB \end{array}$$ (11)

The range of the ROC curve is from 0 to 1. The symbol d represents a small change and measures how much the A changes as the B changes.

## System analysis

This section comprises experimental setup, evaluation metrics, experimental findings, and lastly, discussion.

### Experimental setup

The experimental setup for this study aims to fine-tune MobileBERT for SA and to include methods, i.e., knowledge distillation and data augmentation, in order to enhance the model performance. The setup starts with the installation of required libraries such as optuna for hyper parameter optimization, panda for dealing with dataset, torch for model building. A custom dataset class is implemented, Review Dataset, with MobileBERT’s tokenizer for transforming the text input into tokenized input. The text data is further augmented with synonym replacement from the WordNet corpus to introduce variability in the training data to boost the model’s ability to generalize. Random-based selection of synonyms from the WordNet synsets is performed using synonym-augmentation function.

The main model architecture is based on the MobileBertModel from the Hugging Face transformers library. The model is first used as a backbone in the SimCLR-MobileBERT-model for the pretraining using contrastive learning. Subsequently, FineTuned Sentiment Model is fine-tuned with sentiments and knowledge distillation is used for transferring knowledge from a SimCLR-MobileBERT-Model to FineTunedSentimentModel. The distillation loss, which is implemented in the DistillationLoss class, is a integration of cross-entropy-loss and Kullback KL divergence which works to match the soft targets (teacher’s predictions) and hard targets (true labels). AdamW optimizer for the training process, get-linear-schedule-with-warmup using StratifiedKFold from sklearn.model-selection to maintain consistency of classes in each fold has been performed. Evaluations of a model is performed with different evaluation metrics.

## Results and discussions

The effectiveness of the model was tested on a five-fold cross-validation with the confusion matrices, Precision-Recall (PR) curves, Average Precision (AP) scores, and Principal Component Analysis (PCA) as measures of its effectiveness. These assessment techniques can be used to analyze the learning process of the model in detail, its capacity to generalize, and its capacity to negotiate the trade-off between accuracy and recall as training progresses.

**Fig 2:**
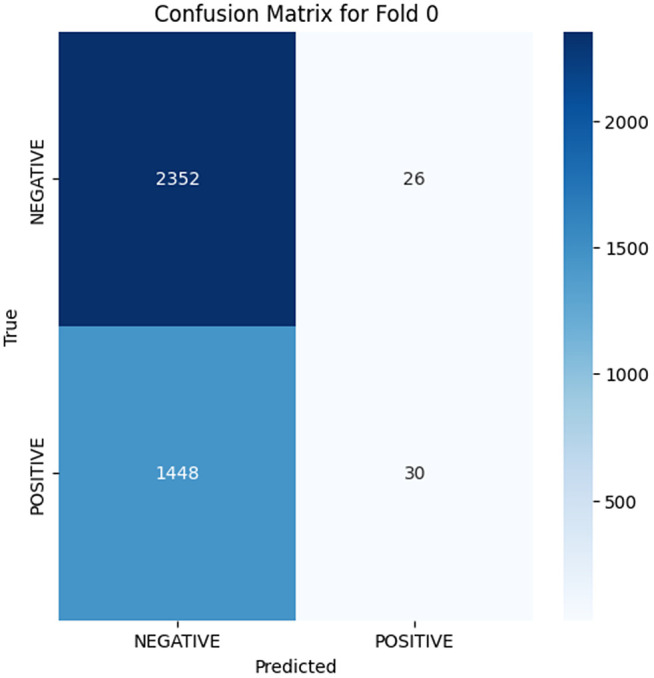
Confusion Matrix for Fold 0.

**Fig 3:**
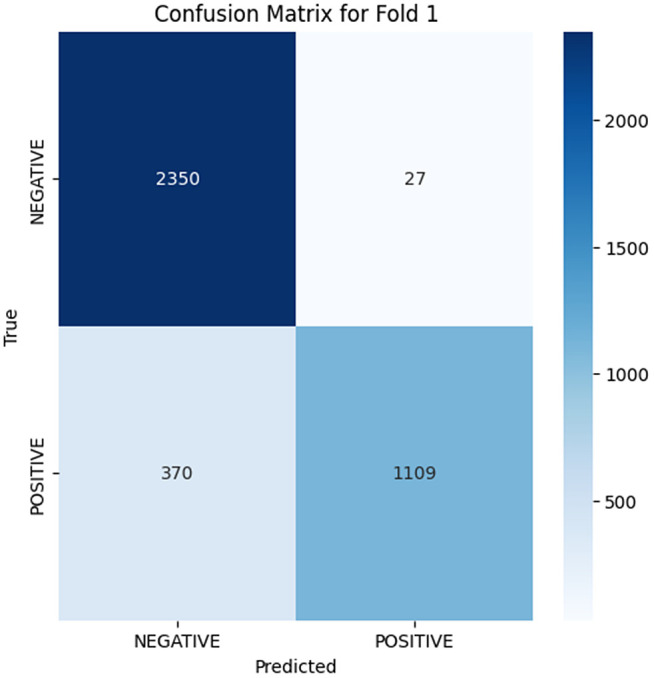
Confusion Matrix for Fold 1.

**Fig 4:**
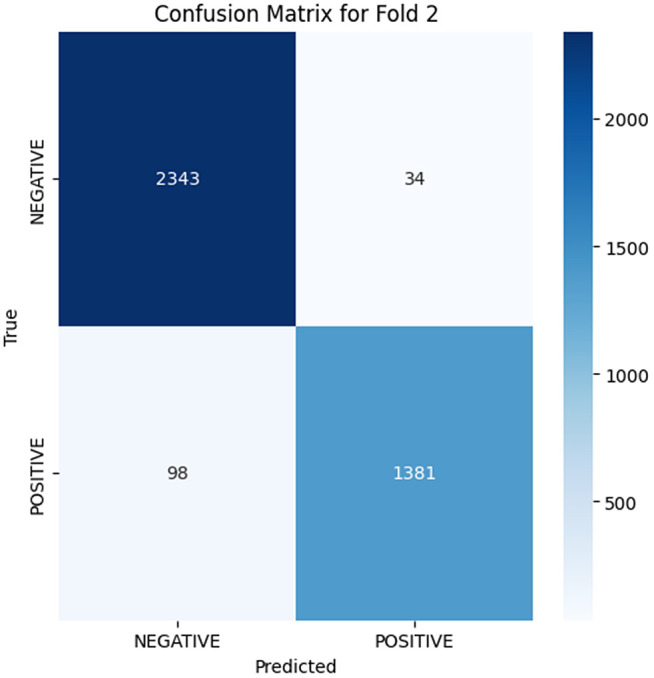
Confusion Matrix for Fold 2.

**Fig 5:**
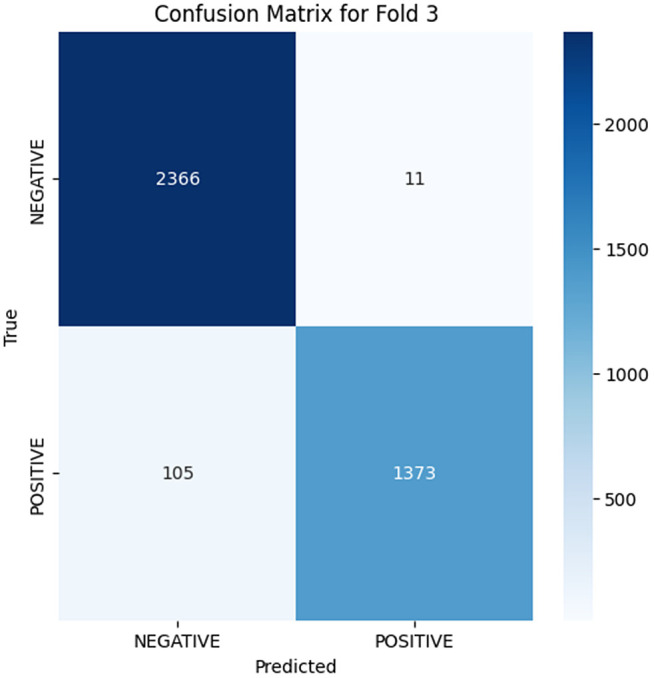
Confusion Matrix for Fold 3.

**Fig 6:**
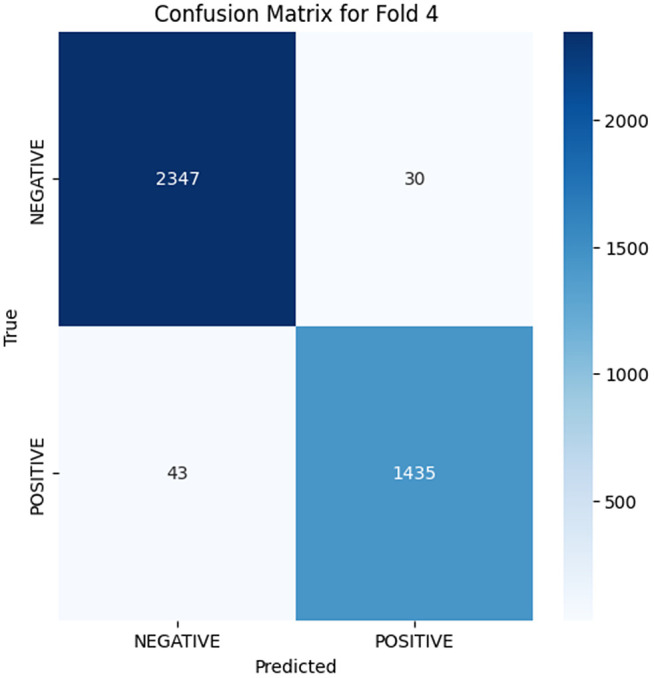
Confusion Matrix for Fold 4.

The analysis of the confusion matrix through the folds demonstrates an increase in the performance of the model during the classification. AP is a metric used to summarize the PR curve by computing the area under it. The gradual increase in the score of AP of the folds indicates that the model becomes more specialized in enhancing the decision boundaries that are increasingly capable of accommodating the class differences and can make generalizations more accurately in the circumstance of unseen data. The precision-recall curves on the different folds, as illustrated in Figs 2–6, show a significant increase in the model’s being able to balance the precision and recall and increase as it goes through the cross-validation.

### Fold 0 Results

In Fold 0 in Figs 2–6, there were high model accuracy in 2352 true negatives and 1448 false negatives, but the number of misclassifications (26 false positives and 30 true-positives) reveals the discrimination of the classes. The PR curve (AP = 0.4432) in Fold 0 exhibits a very steep Precision vs. Recall drop, which is strongly influenced by a high rate of False-Positives attempting to recall more samples of the positions, which is an inefficiency of the model itself at the start of the Fold.

### Fold 1 Results

**Fig 7:**
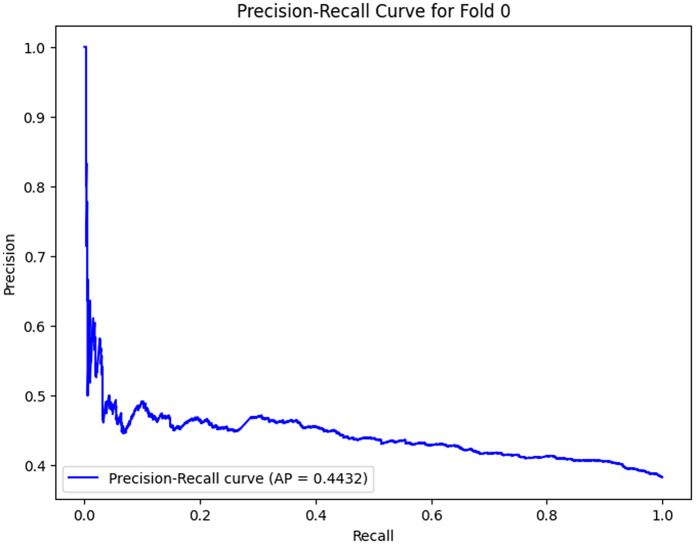
Precision–Recall Curve for Fold 0.

**Fig 8:**
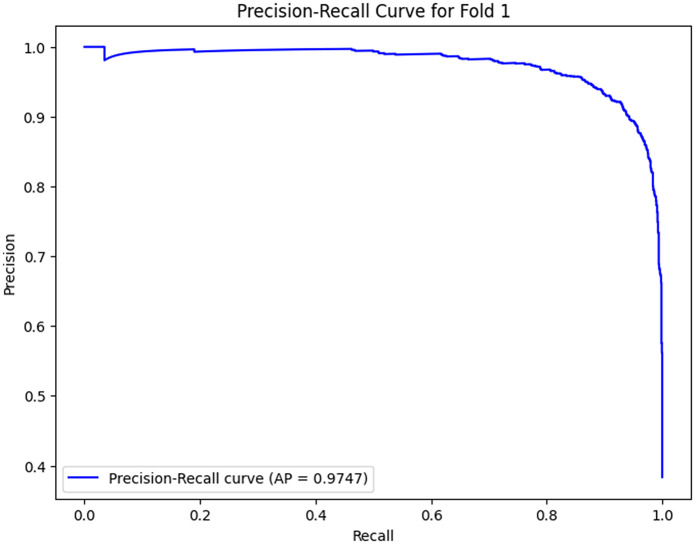
Precision–Recall Curve for Fold 1.

**Fig 9:**
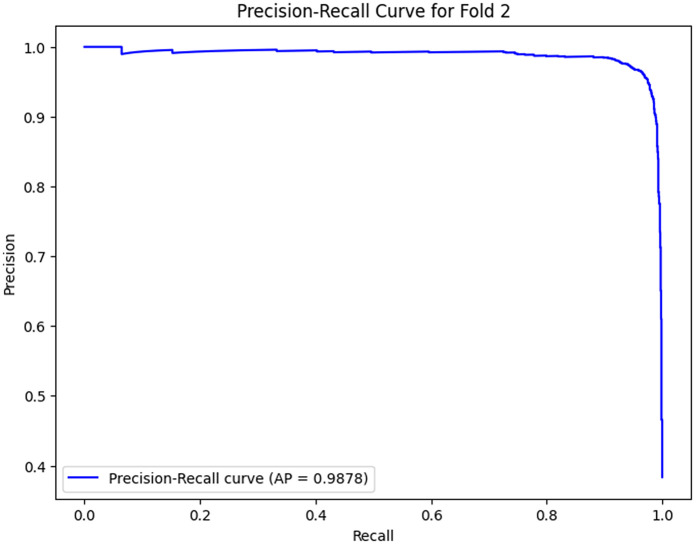
Precision–Recall Curve for Fold 2.

**Fig 10:**
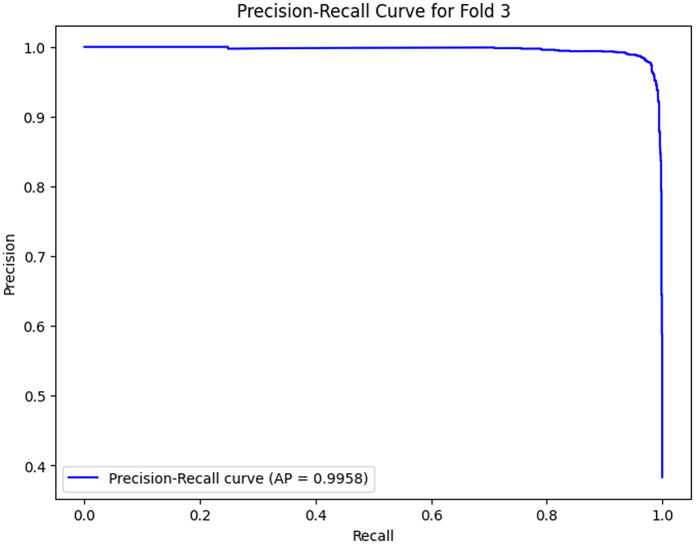
Precision–Recall Curve for Fold 3.

**Fig 11:**
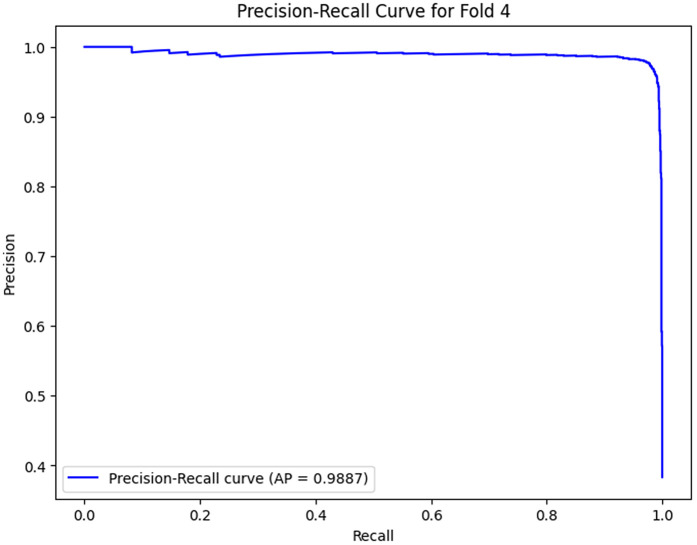
Precision–Recall Curve for Fold 4.

As the model advances to Fold 1, the performance is enhanced; the AP score goes up to 0.9747. The confusion matrix reveals 2350 true negatives and 1109 true positives, with 27 false positives and a significantly high number of false negatives (370). The model remembers more positive samples at the expense of a higher false negative rate.‌‌ Figs 7–11 below shows the PR curve of this fold (AP = 0.9747), demonstrating a defined tradeoff between precision and recall, where accuracy is still comparably high at the start of the recall values but begins to decrease as recall rises. This indicates that the model has not maximized its classification ability, but the point is that it is moving in the right direction of maximizing recall at the expense of precision, which is a familiar attribute in the early stages of learning.

### Fold 2 Results

A more fined learning process is exhibited in Fold 2 where the model is depicted to be very refined. The confusions matrix depicts that 2343 negatives were true and 1381 were true positives, this is much less than before, all those false negatives have been reduced, and this means that the model is now more precise in finding positives without compromising too much precision. The AP score of 0.9878 demonstrates a significant increase of the precision and recall, and the PR curve displays the more fluent decay, which is much more appropriate to regulate the trade-off between the two indicators. These features make the model reasonably learn, improve on the decision boundaries, and also learn to get more discriminative features out of the training set at this stage.

### Fold 3 Results

At Fold 3, the model achieves the stability stage of the performance, as did the AP score 0.9958. The confusion matrix reveals that there are 2366 true negatives and 1373 true positives, although the amount of false positives (11) and false negatives (105) is very minor. The PR curve and AP score of 0.9958 indicate there is a near-perfect trade-off between precision and recall, where the model stands at a high level of precision throughout the entire range of recall. This shows that the model has acquired the skills to determine positive and negative instances effectively without compromising a substantial amount of precision.

### Fold 4 Results

The highest level of performance of the model is in Fold 4 with a value of 0.9887 on AP. The confusion table depicts the true negatives and true positives are 2347 and 1435, respectively, with very few misclassifications (43 false negatives and 30 false positives). The PR curve and (AP = 0.9887) in this fold depict a close balance in precision and recall, signifying that the model has now attained high levels of generalization, and it can now equally detect a positive or negative instance. The diminishing false positives and false negatives as compared to the previous folds indicate that the model has narrowed its decision boundaries, which results in the reduced number of misclassifications.

Folds 0 to Fold 4 are represented as Figs 2–6 while PR curves of Fold 0 to Fold 4 are shown as Figs 7–11, respectively.

### ROC analysis of each fold

**Fig 12:**
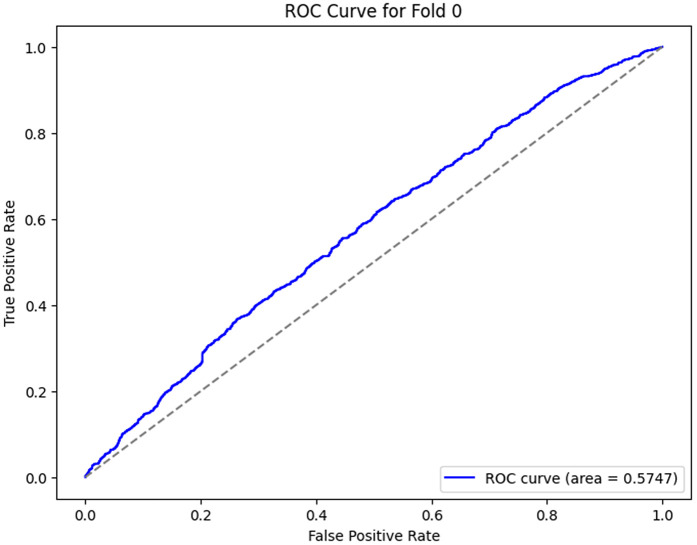
ROC Curve for Fold 0.

**Fig 13:**
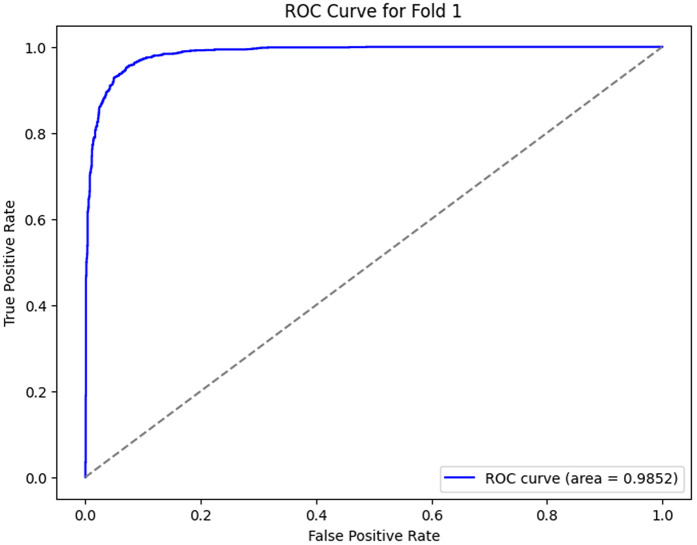
ROC Curve for Fold 1.

**Fig 14:**
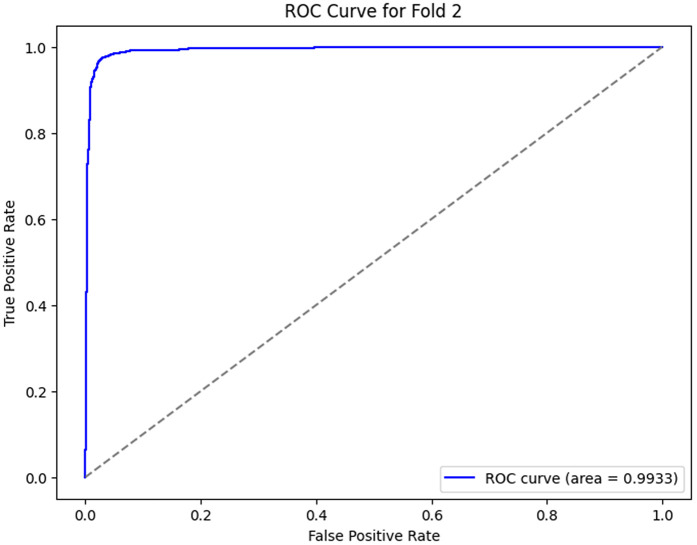
ROC Curve for Fold 2.

**Fig 15:**
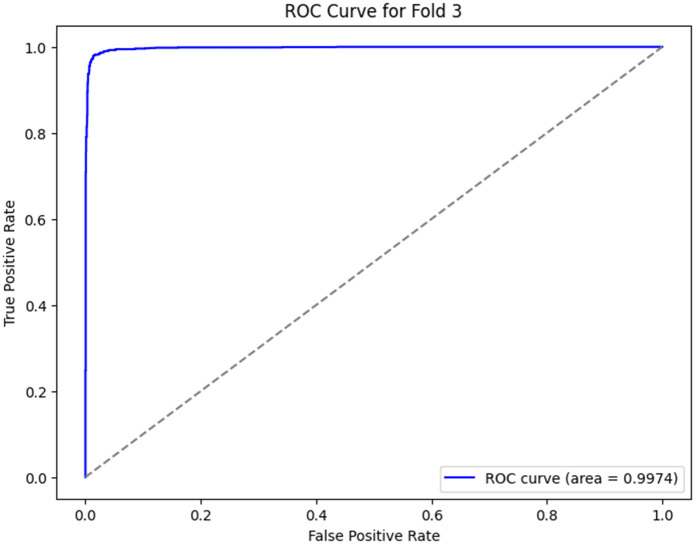
ROC Curve for Fold 3.

**Fig 16:**
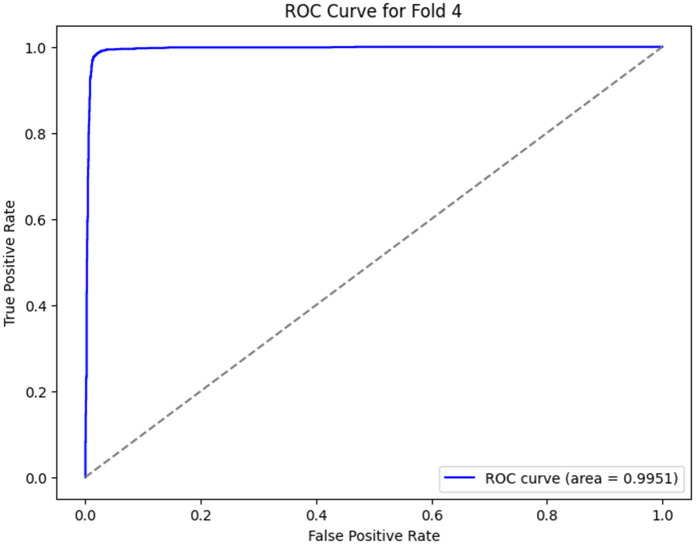
ROC Curve for Fold 4.

The ROC curves, as shown in Figs 12–16 for the different folds, suggest that the model becomes more and more successful at differentiating between the positive and negative classes with increasing Area Under the Curve (AUC) values as we go all the way from Fold 0 to Fold 4. Fold 0 (AUC = 0.5747) has a relatively poor performance, with the curve being a little bit better than the diagonal line, which means that the model does slightly better than random guessing. On the opposite side, Fold 1 (AUC = 0.9852), Fold 2 (AUC = 0.9933), Fold 3 (AUC = 0.9974), and Fold 4 (AUC = 0.9951) exhibit a much stronger result, showing a curve closer to the top-left corner, which is a high positive class with very few false-positives. This sharp increase in the curves indicates that the model’s performance in classifying both positive and negative instances improves as the folds go by, with Fold 3 showing almost perfect classification. These results represent the model improvement process, whereby it is trained over the cross-validation process; the model decision boundaries are iteratively improved, and the misclassifications are minimized. The rising values of every fold are an indication that the model is learning and adapting to the data successfully for classification accuracy and generalization improvement.

### PCA of each fold

**Fig 17:**
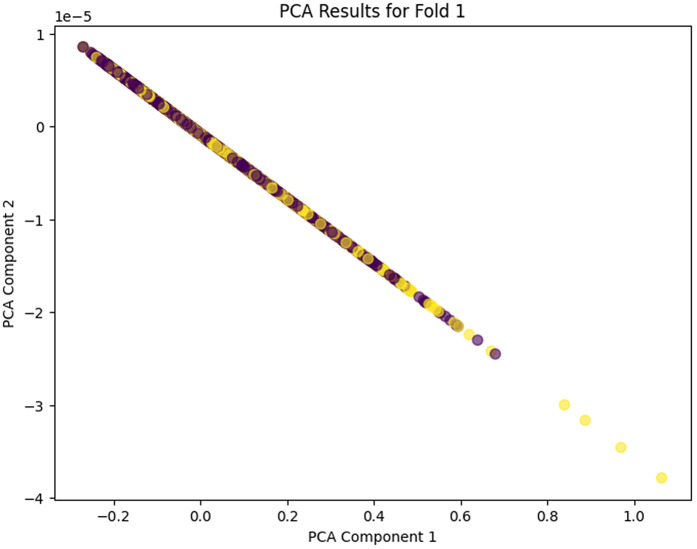
PCA Results for Fold 0.

**Fig 18:**
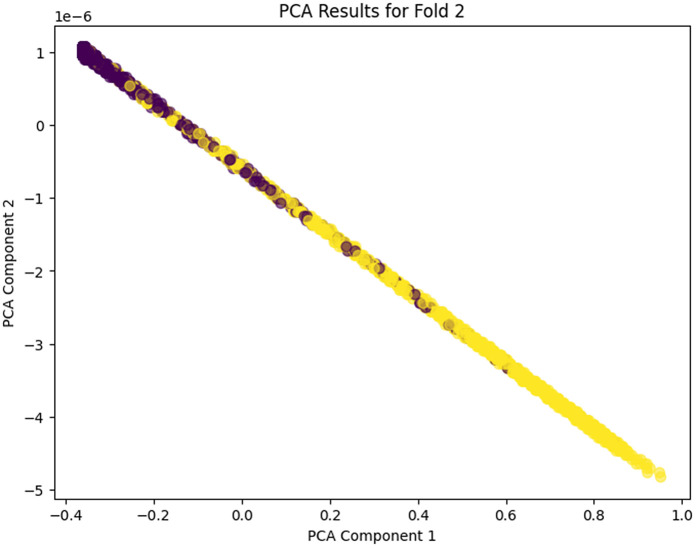
PCA Results for Fold 1.

**Fig 19:**
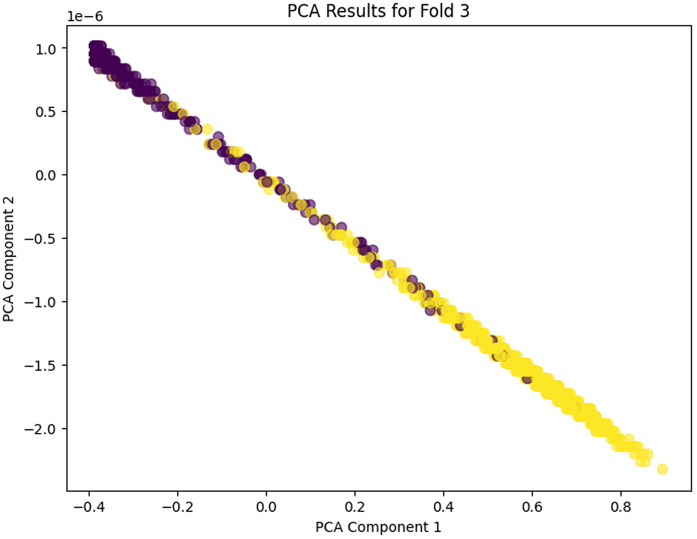
PCA Results for Fold 2.

**Fig 20:**
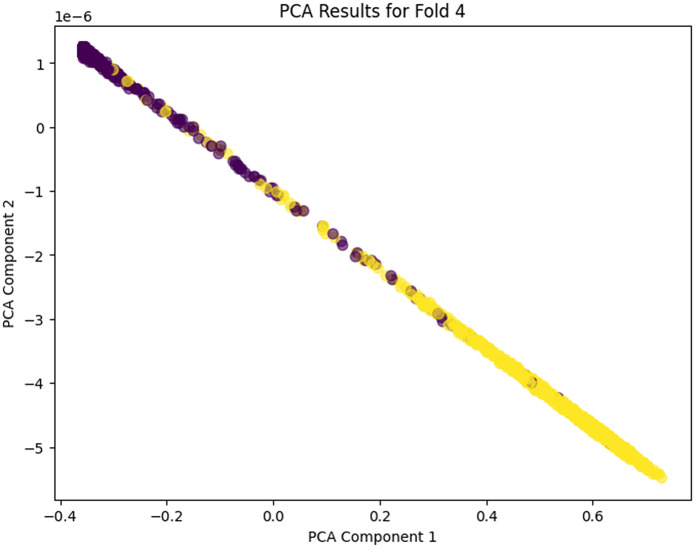
PCA Results for Fold 3.

**Fig 21:**
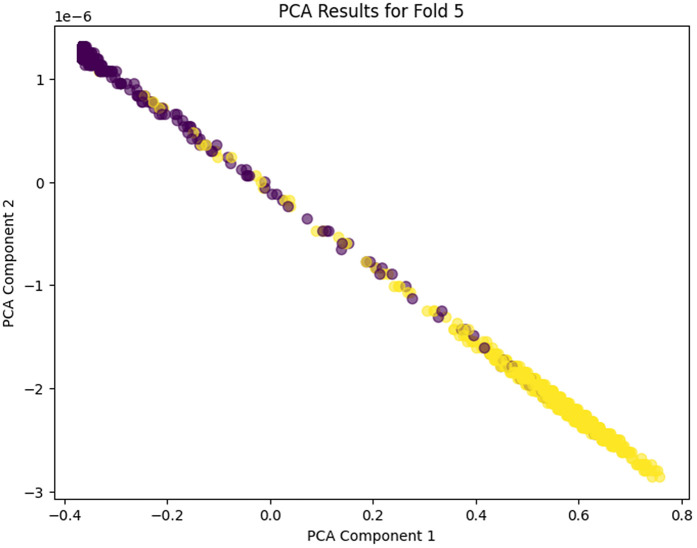
PCA Results for Fold 4.

The PCA analysis across the five folds does clearly show the progressive learning and generalization ability of the proposed model. In PCA Fold 0, the data points are pressed near one dominating direction, meaning that poor variance capture results, and the feature representation in this first stage is more limited. Moving to PCA Fold 1, there’s an increase in the spread along PCA Component 1, indicating an increase in the capture of variance and an increase in sensitivity to inherent differences in the features. In PCA Fold 2 the distribution is more balanced between the components, which would indicate that the model is learning more discriminative and stable feature representations. PCA Fold 3 has a relatively compact but well-structured distribution, which seems to reduce noise as well as provide better, consistent feature extraction. Finally, PCA Fold 4 is the most mature representation with the coherent separation and control of the dispersion in the main principal components, which shows the maturity and strength of the model in describing the essential nature of the data. In this case, the gradual acceleration from Fold 0 to Fold 4 indicates that the proposed model has learned to learn better features, variance, and generalization ability among cross-validation folds. PCA Fold 0, PCA Fold 1, PCA Fold 2, PCA Fold 3, and PCA Fold 4 results are illustrated in Figs 17–21.

The model evaluation result by each fold is shown as Table 4.

**Table 4:** Model evaluation results across five-fold cross-validation.

Fold #	Accuracy (%)	Precision (%)	Recall (%)	ROC–AUC (%)
0	61.77	53.57	2.03	57.47
1	89.70	97.62	74.98	98.52
2	96.58	97.60	93.37	99.33
3	96.99	99.21	92.90	99.74
4	98.11	97.95	97.09	99.51
**Average Score**	**88.63**	**89.19**	**72.07**	**90.91**

### Comparative analysis with related literature

In comparison to different models that exist in the literature, our proposed model categorizes several advanced techniques that increase the level of accuracy, semantic understanding, and generalization for different datasets. For instance, in [13], while the precision and recall of AdaBoost, XGBoost, and ANNs were 98% and 85%, respectively, they didn’t have advanced techniques to extract features and detect complex semantic features. Our proposed model goes beyond this by incorporating SimCLR for self-supervised learning for better feature extraction and MobileBERT for ensuring computational efficiency and excellent generalization across different data distributions. Similarly, the work presented in [14] identifies the importance of app store data for research but does not utilize the SA. Our model fills this gap by using knowledge distillation and data augmentation, which helps improve both accuracy and semantic understanding of the classification and better covers user sentiments. In [15], the UX-MAPPER tool, although very useful to comprehend the UX factors, lacks the addressing of SA capabilities. Our proposed model is an improvement over this, and it uses something like MobileBERT and SimCLR that helps in performing better SA and promoting more semantic understanding of users’ feedback, which can provide more actionable insights to app developers. In [16], although SVM outperformed NB on classifying sentiment, there is a big advantage of using MobileBERT and SimCLR on our decision-making model, not only improving the feature extraction but also improving the capability on diverse datasets, making it not only capture the semantic nuances in user reviews. IndoBERT in [17] achieved 87.76% accuracy using a dictionary-based method, but our model goes beyond a dictionary-based approach using MobileBERT for efficiency, SimCLR for self-supervised learning, and SHAP for interpretability, which provides superior semantic analysis and generalization. The fine-tuning of BERT in [18] showed an F1 score of 0.80 for positive sentiment; however, our model, by using MobileBERT and SimCLR, outperforms BERT in extracting better features and providing semantic understanding to achieve better generalization among datasets. In [19], SVM achieved an accuracy of 84% for sentiment classification, whereas our model achieves an accuracy and a ROC AUC of 88.63% and 90.91%, respectively, demonstrating even better generalization and semantic processing capability using MobileBERT, SimCLR, and data augmentation. The study in [20] suggests Wiscom gives valuable suggestions but lacks SA capabilities, whereas our model, through MobileBERT and SimCLR, provides full-fledged SA, providing more accurate and semantically enriched insights into user feedback. In [21], BERT achieved positive sentiment percentages for different categories of apps, but our model improves upon BERT using MobileBERT for efficiency, SimCLR for feature extraction, and knowledge distillation for better generalization, achieving better semantic analysis. In [22], feature selection methods, such as Information Gain, GINI Index, and Correlation Matrix, help SVM to improve its performance. Our proposed model outperforms this in that it incorporates PCA for dimensionality reduction and SimCLR for better feature extraction, which helps in getting better results in both accuracy and semantic understanding. Word2Vec and LSTM in [24] helped recall a high accuracy rate; however, there are modes lacking the flexibility and semantic depth in our model by integrating MobileBERT and SimClar and data augmentation that perform better on diverse datasets. In [27], though SA on mobile app service quality has been conducted, it did not use the advanced method, such as SimCLR and MobileBERT, that our model uses to help the accuracy and robustness of user feeds and understand semantic information. The research study in [29], which focuses on the SA in the context of m-banking, does not use self-supervised learning and advanced transformers, which are the foundation of our model for ensuring better generalization, scalability, and semantic analysis. In [32], ABSA with BERT was used for the sentiment triplet extraction, but our model is improved in terms of SimCLR for feature extraction, MobileBERT for efficiency, and knowledge distillation for better robustness and semantic understanding. SVM in [34] achieved 89.33% accuracy but lacks semantic capabilities as compared to our proposed approach and provides better generalization, accuracy, and semantic knowledge among various review data. Finally, in [42], different ML algorithms, such as RF, were applied, but our model is superior to these techniques in terms of MobileBERT and SimCLR in terms of feature extraction, accuracy, and semantic variations in reviews. This makes our proposed model more effective in extracting insights and capturing the complexity of the semantics of user feedback than traditional ML approaches.

### Comparative analysis with baseline approaches

Compared to the baseline approaches, which are mainly based on traditional ML models or lightweight transformer-based frameworks such as DistilBERT, our model builds on the recent state-of-the-art algorithms like SimCLR and MobileBERT, which contribute significantly to the ability of the models to understand the semantics and extract features. Nakamura et al. (2025) [15] were interested in automated UX review analysis, but they did not implement formal sentiment classification, whereas our proposed model uses the SimCLR contrastive learning algorithm and MobileBERT to extract sentiments efficiently, which helps to interpret the emotion on a deeper level. In the same manner, feature selection and topic modeling are used by Salleh et al. (2025) [23] and Susanto and Mauritsius (2025) [24], but they are not sufficient to provide the deep semantic representation, which is solved by our model through combining SimCLR with MobileBERT and PCA, thereby improving the generalization. On the contrary, Jafari et al. (2025) [25] are more concerned with prioritizing reviews to action by developers, but their model is less optimized with regard to sentiment classification and context. Moreover, the methods used by authors in [38,39] are limited to DistilBERT and classical text mining tools that have a smaller semantic capacity and cannot effectively handle complex linguistic phenomena. Amirkhalili and Wong (2025) [40] and Rahayu and Hasibuan (2025) [42] reviewed banking apps but did not employ self-supervised learning or advanced transformers in comparison to our proposed model, which offers better contextual insights, model accuracy, and practical viability by combining SimCLR with MobileBERT. A comparative analysis with the baseline approaches is shown in Table 5.

**Table 5:** Comparative analysis with baseline approaches.

Reference	Model/Technique	Key Features	Limitations	Proposed Model Contribution
[15] Nakamura et al. (2025)	UX-MAPPER	Automated UX review analysis	No formal sentiment classification	MobileBERT + SimCLR for deep semantic sentiment extraction
[22] Salleh et al. (2025)	Feature Selection Methods	Improves classification using feature selection	Limited contextual understanding	SimCLR + MobileBERT + PCA for deep features and noise reduction
[24] Susanto & Mauritsius (2025)	Sentiment + Topic Modeling	Combines sentiment, topic modeling, summarization	Misses nuanced sentiment	SimCLR + MobileBERT for richer semantic representation
[25] Jafari et al. (2025)	Review Prioritization Model	Ranks reviews for developer response	Not optimized for sentiment accuracy	MobileBERT + SimCLR + augmentation for accurate sentiment detection
[34] Prasetyaningrum (2025)	ML-based Classification	Uses traditional ML classifiers	Weak semantic understanding	MobileBERT + SimCLR + distillation for contextual learning
[38] Ullah et al. (2025)	DistilBERT Model	Lightweight transformer for sentiment analysis	Limited representation capacity	MobileBERT + SimCLR for richer feature learning
[39] Khan & Khan (2025)	Text Mining + SA	Classical sentiment analysis approach	Fails on complex language patterns	MobileBERT + SimCLR + SHAP for deep semantic analysis
[40] Amirkhalili & Wong (2025)	Statistical Text Analysis	Combines statistical and text analysis	Not transformer-based	SimCLR + MobileBERT for contextual learning
[42] Rahayu & Hasibuan (2025)	ML-based SA Models	Uses multiple ML algorithms	Lacks deep semantic analysis	MobileBERT + SimCLR for improved accuracy and semantics

### Threats to validity and limitations

This study has several limitations. First, the subjective judgment can still be operationalized in the manual categorization of mobile app reviews with performance parameter-based guidance of appraisal theory, as well as the use of SHAP-based interpretability. Second, the sample of reviews in mobile apps is also narrow and this could limit the extrapolation of the results to other ypes of user-generated content. Third, linguistic variability in synonym-based augmentation with WordNet can also present the problem of linguistic variation that is not necessarily representative of natural user queries. Fourth, despite the fact that MobileBERT and SimCLR are efficient and representation learning models, the performance of the models can fluctuate when used on unseen datasets with a varying linguistic pattern. Lastly, stratified k-fold cross-validation, Optuna optimization, and distillation enhance robustness, but these methods cannot remove the threats of dataset bias, overfitting, or domain dependency.

## Conclusion and future work

This study introduces a complete and powerful framework for the SA of mobile apps reviews by integrating several advanced techniques, including SimCLR for self-supervised feature extraction, MobileBERT, a hybrid DL-based approach for efficient sentiment classification, and knowledge distillation for better model generalization. The integration of SHAP provides interpretability, which becomes an important part of explainable AI in SA. The proposed approach deals with linguistic diversity through data augmentation and is optimized by the Optuna method, and the resultant model achieved 88.63% mean fold accuracy and 90.91% ROC AUC. The proposed model not only is able to improve the performance of sentiment classification, but it also provides a scalable and efficient solution for real-world applications, which can handle an imbalanced dataset and computational efficiency.

Future work should investigate the incorporation of additional advanced data augmentation techniques, multi-modal learning for better understanding the context, and domain adaptation techniques for further improvement on the generalization of the model across different industries. Furthermore, deployment and performance monitoring of the system in real time on various platforms would be useful to understand its robustness and scalability in a real-world scenario.

## Supporting information

S1 Table [45]. Dataset. (CSV)
